# New Max Verstappen’s on the Rise?: Go-Kart Incidents in a Level-I Trauma Center in the Netherlands

**DOI:** 10.3390/children12111492

**Published:** 2025-11-04

**Authors:** Rania Farkhani, Elisa G. Hamer, Erik Hermans, Manouk Backes, Stijn D. Nelen

**Affiliations:** 1 Nijmegen Collaborative Research Consortium on Pediatric Trauma (NCRC-PT), 6525 GA Nijmegen, The Netherlands; 2 Department of Pediatric Neurology, Radboud University Medical Center, 6500 HB Nijmegen, The Netherlands; elisa.hamer@radboudumc.nl; 3 Department of Trauma Surgery, Radboud University Medical Center, 6500 HB Nijmegen, The Netherlands; erik.hermans@radboudumc.nl (E.H.); stijn.nelen@radboudumc.nl (S.D.N.); 4 Department of Pediatric Surgery, Radboud University Medical Center, 6500 HB Nijmegen, The Netherlands; manouk.backes@radboudumc.nl

**Keywords:** go-karting, go-kart injuries, pediatric trauma

## Abstract

**Background**: Go-karting has become an increasingly popular motorsport and leisure activity among children of all ages. However, go-karting is not without risks. The main purpose of this study was to assess the number of pediatric patients presenting at the emergency department of a level-I trauma center following a go-kart-related injury event over a nine-year period. Additionally, this study aimed to provide a comprehensive overview of patient characteristics, trauma mechanisms, injury types and use of safety devices. **Methods**: A retrospective single-center cohort study identified all patients that presented at the emergency department from January 2015 to December 2023. Data from the Dutch Nationwide Trauma Registry and medical files were assessed, descriptive statistics were conducted, and cohorts—defined by date—were compared: the first cohort from January 2015 to December 2021, and the second from January 2022 to December 2023, with December 2021 marking the moment when Max Verstappen became the first dutchman to win the Formula One World Driver’s Championship. **Results**: A total of 14 patients were identified, with an incidence rate of respectively 0.86 and 4.0 per year for the cohorts. In the total study population, 86% were male and the mean age was 12 years (range 4–17). Collision was the most common trauma mechanism with velocities even exceeding 70 km/h. Reported use of a helmet and seatbelt were respectively 64% and 29%. The mean Injury Severity Score (ISS) was 8.6 (±8.6). 57% of the patients encountered minor injuries (ISS ≤ 8), encompassing mostly soft tissue injuries, whereas truncal injuries occurred most frequently in moderately (ISS 9–15) and severely (ISS ≥ 16) injured children. **Conclusions**: This study has found that although the number of cases was relatively low, an increase in injuries was seen after 2021 in go-karting in children. Almost half of patients were seriously injured, requiring admission at the least. Resulting from this, considerations on minimum age for participation, enforcement of the use of safety devices including additional safety measures targeting truncal protection, and more clear laws and regulations are advised.

## 1. Introduction

On 12 December 2021, Max Verstappen became the first Dutchman to win the Formula One World Drivers’ Championship. Ever since, go-karting has become an increasingly appealing leisure activity and motorsport among children in the Netherlands. Children aspire to become the next Max Verstappen on the circuit [1].

Despite the exhilarating experience, the risk of injury associated with operating go-karts remains a concern. These risks can be attributed to several factors, including high velocity in go-karting, a relatively unprotected trunk and extremities while seated in a go-kart, lack of experience, underutilization of safety devices, and limited formal instructions.

Literature on the risks of go-karting dates back to the 1970’s. Miller et al. observed 12 cases of severely injured patients within a three month period at a single fairground [2]. Most injuries were caused by blunt trauma from the steering wheel and rapid deceleration, and could have been prevented or reduced in severity with the use of a seatbelt. In 1978, Youngson and Baker described the number (*n* = 177), types, and mechanism of injuries over a four-year period at a single kart track. Modifications taken to reduce the incidence and severity of injuries were also discussed [3]. These included the use of helmets and seatbelts, whereas nowadays these are considered mandatory.

Large population studies regarding go-kart-related trauma mechanisms and injuries remain limited, particularly in the Netherlands [4,5,6]. The existing literature primarily consists of case studies, which illustrate the variety of injuries resulting from go-kart incidents [7,8,9,10,11,12,13]. No consistent association with typical injury types has been found. A proposed explanation is the broad spectrum of trauma mechanisms involved in go-karting, ranging from entanglement to blunt force trauma of the steering wheel and collisions. Moreover, one study conducted in a level-I trauma center in the Netherlands continued linking trauma mechanisms to injury patterns through analysis of the sport by an expert panel consisting of three surgeons, with the aim to improve safety measures, injury recognition, and treatment optimalization [7].

This study aims to provide more insight into go-kart-related injuries in pediatric patients focusing on the patients characteristics, trauma mechanisms, injuries and preventive measures. Furthermore, the purpose of this study was to examine the influence of the growing popularity and recognition of go-karting on the number of incidents, as well as on patient characteristics and injuries.

## 2. Methods

### 2.1. Study Design

A retrospective single-center cohort study was conducted to identify all patients younger than 18 years of age who presented at the emergency department (ED) of the Radboudumc following a go-kart-related injury event in the period from 1 January 2015 to 31 December 2023. The Radboudumc is one of the 11 level-I trauma centers in The Netherlands since the regionalization of the Dutch trauma care system in 1998, and serves approximately 1.3 million inhabitants in the eastern region of the country [14]. Within the region, nearly 10 go-kart tracks are located out of the approximately 50 tracks in the country [15].

### 2.2. Study Population and Eligibility Criteria

Patients that presented at the ED following go-kart-related injury events were identified by searching electronic patient records from the period January 2015 to December 2023 on the terms “kart” and “karten” using the software program CTque (IQVIA; version 4.13.1; 2025) [16]. The search results were validated through manual review of the electronic patient records by two researchers. Exclusion criteria were age older than 18 at time of the incident and presentation at the ED unrelated to a go-kart-related injury event. Patients were included regardless of whether or not they had been admitted to the hospital following examination at the emergency department, in order to provide a comprehensive representation of the various injury types and severity. Subsequently, the study population was divided into two cohorts based on the admission period: from 1 January 2015 to 31 December 2021, and from 1 January 2022 to 31 December 2023. Thereby, the cohorts were defined as before and after the Formula One World Championship in 2021, marking a rise in the popularity of go-karting in The Netherlands.

### 2.3. Primary and Secondary Outcomes

The primary outcomes for this study were the number of go-kart-related injury events, as well as injury type and severity. Secondary outcome measures included age, trauma mechanisms, and safety measures. Moreover, the impact of the rise in popularity of go-karting on the number of injury events presenting at the emergency department was explored.

### 2.4. Data Collection

Data were retrieved from the Dutch Nationwide Trauma Registry (DNTR) and medical files. The DNTR is a prospective database which includes all trauma patients that have been admitted to the hospital through the emergency department [17]. Pre-injury variables that were collected from the DNTR include patient characteristics (age at time of the incident and gender) and the American Society of Anesthesiologists (ASA) score [18]. Furthermore, the trauma mechanism (blunt or penetrating) and injury variables including the abbreviated injury scale (AIS), injury severity score (ISS), and Glasgow Coma Scale (GCS) at arrival at the ED were collected [19,20,21]. Treatment variables included the total number of surgical interventions, length of stay on the ward, admission to Intensive Care Unit (ICU) and the total length of stay (LOS). To assess functional outcomes, the Glasgow Outcome Scale (GOS) at hospital discharge was obtained from the DNTR [22].

Data from the DNTR could not be retrieved for all patients due to patients not being admitted to the hospital or transferred to another hospital, and therefore not being included in the national trauma registry. In these cases, data were retrieved from medical records in accordance with the current definitions and as used in the DNTR to minimize missing data [23,24,25].

Data from the DNTR was further supplemented with data from medical records or collected through telephone interviews to further minimize missing data, such as trauma mechanism, impact in km/h, use of safety devices and driving experience. Patients were considered experienced in case of participation in formal trainings and competitions. Inexperience was defined as first time go-karting and go-karting as a leisure activity.

The variable set for this study was established by three researchers. Subsequently, data collection was predominantly conducted by one researcher.

This study was exempted from ethical review board approval.

### 2.5. Statistical Analysis

Continuous variables were presented using medians and interquartile ranges (IQR 25–75th percentile). Categorical variables were presented using frequencies and percentages. Descriptive statistics were performed to characterize the study population. The Shapiro–Wilk test was used to determine the distribution of data within the cohorts. Independent samples *t*-testing for normal distributed continuous data (with equal variances) and Fisher’s exact testing for dichotomous categorical data were used for comparisons between the cohorts. Non-parametric tests included Mann–Whitney U testing for continuous data and Chi-square testing for nominal/ordinal data. *p*-values < 0.05 were considered statistically significant. With the purpose of correcting for multiple testing, the Bonferroni correction was used to determine an adjusted significance level (αadjusted = 0.005). All statistical analyses were performed with the SPSS statistical software program (version 29.0: IBM, Armonk, NY, USA). Due to the retrospective nature of the study, missing data were inevitable despite the efforts to minimize this. Missing data were labeled as unknown and indicated in the results section accordingly, as well as inclusion in analysis. Other methods for handling missing data such as imputation or listwise deletion were not used due to the sample size.

## 3. Results

### 3.1. Patient Characteristics

From January 2015 to December 2023, 30 patients were initially identified with CTque, of which 14 met the eligibility criteria and were included in the study. The majority of patients were male (86%) and the mean age was 12 years (SD ± 4 and range 4–17 years). Of all patients that presented at the emergency department following a go-kart-related injury event it is known that five children were experienced drivers, i.e., participated in formal trainings and competitions, of which the youngest was four years old. With regards to the use of safety devices, helmet use was most commonly reported (*n* = 9, 64%), followed by the use of protective clothing (*n* = 5, 36%), neck brace (*n* = 5, 36%) and seatbelt (*n* = 4, 29%). In three cases, no seatbelt was explicitly reported due to dysfunction or the go-kart not being equipped with a seatbelt. In two cases, use of safety devices remained entirely unknown. Blunt trauma was encountered for all cases. Trauma mechanisms involved an interplay of events, which should be considered when interpreting the results separately. Abrupt deceleration caused by collision of the go-kart with a wall, tire-stack, or safety barrier occurred in most patients (*n* = 9, 64%), followed by impact trauma from the steering wheel (*n* = 5, 36%) and seatbelt (*n* = 3, 21%), fall from the vehicle (*n* = 3, 21%) and overturning (*n* = 3, 36%). One patient was struck by a go-kart and suffered from entanglement of a finger in the motorcycle chain (Table 1).

### 3.2. Primary and Secondary Outcomes

The number of patients presenting at the emergency department following a go-kart-related injury event remained consistent with one case yearly until 2023, and none reported in 2017. In 2023, there was an increase, with seven reported cases. Overall, injuries varied in type and severity. Minor injuries (defined as an ISS ≤ 8) were most common among patients (*n* = 8, 57.1%) and mainly included soft tissue (skin/subcutaneous/muscle) injuries. These cases further included a single rib fracture without flail, a proximal humerus fracture, partial amputation of digit 3 and a tendon tear of digit 4 following entanglement, and mild traumatic brain injury in one patient.

In moderate (ISS 9–15, *n* = 3) and severely (ISS ≥ 16, *n* = 3) injured patients, abdominal injuries incurred most frequently. Five of these patients (respectively 67% of the moderate and 100% of the severely injured group) suffered from serious abdominal injuries, including complete transection of the duodenum with contusion of the pancreas and colon near the hepatic flexure in one case; contusion of the duodenum; two cases respectively of grade-III and IV liver lacerations; and a grade-IV spleen laceration with a concomitant grade-II liver laceration in one case. Additional injuries were also encountered in these patients with the exception of one patient who suffered from isolated abdominal injuries. These injuries included serious thorax injuries in two cases with bilateral lung contusions and multiple (≥3) rib fractures without flail in one case. Minor and moderate extremity injuries were encountered in two cases and encompassed a scapular body fracture and distal radius fracture Salter Harris type II along with a greenstick fracture of the distal ulna. Severe extremity trauma resulted in a spiral midshaft fracture of the femur with lateral dislocation in one patient with no other injuries. Traumatic brain injury was limited in the study population with a total of two patients encountering mild TBI with loss of consciousness < 30 min and no other abnormalities following imaging (computed tomography). Hypoxic brain damage associated with coma ≥ 24 h occurred in one multi trauma patient secondary to shock from a grade-IV liver laceration. Since this was a secondary effect of the trauma it was not coded within the abbreviated injury scale. Initial imaging with a CT-head showed no abnormalities, and an MRI performed after several days during admission showed a small focus of diffusion restriction in the right cerebellar lobe (Table 1).

### 3.3. Treatment

All patients were examined at the emergency department. Subsequently, half of the patients (*n* = 7) were admitted to the hospital, of which one was at some point transferred to another level-II hospital with serious extremity injury and an indication for surgical intervention. The median LOS in this group (*n* = 6) was 3 days (IQR 17, range 1–41), with a median stay on the ICU of 0 days (IQR 3, range 0–25). The majority of patients were treated non-operatively, whereas two patients required surgical intervention due to serious abdominal injury. The number of surgical interventions ranged from zero to four.

Furthermore, one major trauma patient underwent embolization of the hepatic artery after a rebleed during admission and needed multiple blood transfusions due to hemodynamic instability (Table 2).

### 3.4. Before and After the ’21 World Championship

The rate of new go-kart incident cases per year presenting at the emergency department of the Radboudumc for the cohorts was respectively 0.86 and 4.0 (six cases over a period of seven years versus eight cases in two years). There were no other statistical significant differences between the cohorts given the adjusted significance level (αadjusted = 0.005) and *p*-values, which can be found in Table 3.

## 4. Discussion

In the present study, the absolute number of children presenting at the emergency department following a go-kart-related injury event was found to be relatively low. Nonetheless, this study discloses an increase in cases, with eight patients presenting at the emergency department in 2023. Parallel to this finding, in recent years patients of younger age were seen, of which the youngest was four years old. Of all children presenting at the emergency department following a go-kart-related injury event, nearly half were seriously injured requiring admission and in some cases surgical intervention. Truncal injuries were most common in moderate and severely injured children. Regarding safety measures, helmet use was most frequently reported with remarkably no cases of moderate or severe head injury.

Minor injuries were overall common in this study, namely in 57% of the study population, requiring no admission to the hospital or admission for a short period of time for solely observation. In cases of moderately and severely injured patients, truncal injuries-and mainly abdominal were most common, indicating that additional preventative measures should be aimed at this. In pediatric patients suffering from abdominal injuries, the spleen and liver have been shown to be most frequently affected [26]. This also applies to our study population, which can be explained by the greater risk of injury of the abdominal organs in children due to higher transmission of forces and the relatively large surface of liver and spleen compared to adults [27].

The variability of injuries is illustrated and is in line with previously described case studies, despite the common trauma mechanisms [7,8,9,10,11,12,13]. The spectrum of injuries in our study population is however different from, e.g., the early observations of Youngson and Baker at a single kart track over a four-year period in the 1970’s: of 177 patients, 15 patients were severely injured, requiring major surgery. Further definitions of severe injury and major surgery were not provided. Abdominal and thoracic injuries accounted for respectively 5% and 14%, whereas severe extremity (30%), perineum (26%), and face (25%) injuries were more frequent [3]. Youngson and Baker continued describing modifications aiming to reduce the number and severity of injuries, leading us to a proposed explanation for the differences found. Namely, changes in go-kart and track design (i.e., number of pile-ups, track barriers, position of the steering wheel, etc.) in addition to the implementation and enforcement of proper safety devices such as helmets and seatbelts since.

In our study population, helmet use was reported in more than half of the patients (64%). Furthermore, the occurrence of TBI was limited, especially considering TBI as a major cause of death and disability in pediatric trauma patients [28]. A retrospective analysis of all children 0–16 years hospitalized due to injury from 2009 to 2015 has shown that severe head injuries were present in the majority of children with severe injury after road traffic accidents (RTAs) in The Netherlands. Road accidents are, along with domestic violence, the leading cause of injury in pediatric trauma patients in the country [29]. Helmet use in our study population may have contributed to our findings.

Altogether, this has led to questions regarding go-karting and the current practice. Inclusion of young patients and the injury pattern have raised the question of what age generally should be considered justified. Moreover, the importance of the improvement as well as enforcement of safety standards is emphasized. To our knowledge, laws and regulations at the national level remain unclear and legal disputes questioning liability have been presented over the years [30,31]. Matters such as no minimal age requirement, driving competence, insufficient instructions, and supervision have been questioned. Therefore, legislation with the purpose to achieve uniformity and maintain safety standards is appropriate and could be beneficial for all involved parties: children, parents/caregivers, and track holders. Safety measures with additional truncal protection and minimum age for participation could be starting points for improvement. However, this is from one perspective. Go-karting offers a stimulating experience and is perceived as beneficial in various ways and promoted as such. For instance, proponents of go-karting suggest that it enhances reflexes, situational awareness, concentration, car control, and physique, which may be considered valuable qualities to pursue [32]. Different perspectives and values should be considered when evaluating concerns and problems not only for better understanding, but also to encourage more shared and effective solution thinking, decision making, and commitment.

### 4.1. Strengths and Limitations

The limitations of this study are in line with the limitations that come with a retrospective study. With the aim to minimize incomplete and missing data, some data were retrospectively collected by phone. Despite this, we should be aware of the underestimation of variables, such as the use of safety devices. Furthermore, the study population was limited to patients presenting at our hospital, a level-I trauma center, and thereby representing a certain patient population. It is likely that this patient population is relatively more seriously injured. Additionally, the observed increase in patients presenting at the emergency department following a go-kart-related injury event may be coincidental. Although a connection seems plausible, this study has not proven a definitive link between the increased incidence and Max Verstappen’s Championship in 2021, nor was a study question included regarding this. Lastly, the modest size of the study population has its implications on the power and therefore on the interpretation of results and drawing conclusions. Altogether, this study has provided an overview of available literature, our findings, and identified areas for improvement.

### 4.2. Conclusion and Future Implications

In conclusion, this study has found an increase in the number of injured children presenting at our hospital following a go-kart-related injury event after 2021. Nearly half of the children were seriously injured, reflecting the potential dangers. Areas for improvement have thereby been brought to attention. Resulting from this, considerations on minimum age for participation, enforcement of the use of safety devices including helmet and seatbelt use, additional safety measures targeting truncal protection, and overarching more clear laws and regulations are advised.

## Figures and Tables

**Table 1:** Descriptive characteristics of patients presenting at the emergency department after sustaining a go-kart incident.

Total Study Population (N = 14)	
Age (in years), mean ± SD (range)	11.5 ± 3.7 (4–17)
Gender, N (%)	
Male	12 (85.7)
Female	2 (14.3)
ASA score ^1^, N (%)	
1 Normal health	11 (78.6)
2 Mild systemic disease	3 (21.4)
3 Severe systemic disease	0
4 Severe systemic disease that is a constant threat life	0
5 Moribund patient	0
Driving experience ^2^, N (%)	
Unexperienced	2 (14.3)
Experienced	5 (35.7)
Unknown	7 (50)
Safety devices usage ^2^, N (%)	
Helmet	9 (64.3)
Neck brace	5 (35.7)
Seatbelt	4 (28.6)
Protective clothing	5 (35.7)
Multiple	8 (57.1)
Unknown	2 (14.3)
Impact in km/h ^3^, mean ± SD	58.7 ± 12.5
Shock on arrival ED, N (%)	1 (7.1)
AIS ^1^ ≥ 3 per body region, N (%)	
Head ^4^	0
Face	0
Neck	0
Spine	0
Thorax	2 (14.3)
Abdomen	5 (35.7)
Upper extremities	0
Lower extremities	1 (7.1)
External/other	0
ISS ^1^, mean ± SD (range)	8.57 ± 8.64 (1–25)
ISS, N (%)	
≤8 minor	8 (57.1)
9–15	3 (21.4)
16–24	2 (14.3)
≥25	1 (7.1)

^1^ Abbreviations: ASA American Society of Anesthesiologists score, AIS Abbreviated Injury Scale, ISS Injury Severity Score. ^2^ For two patients, driving inexperience could be determined explicitly from reports and telephone interview. The use of safety devices remained entirely unknown for two patients. ^3^ The mean impact was calculated for eight patients and could not be retrieved for six patients through medical files nor telephone interviews. ^4^ AIS head was coded “9” (which represents unknown severity of injury) in one patient due to hypoxic brain damage secondary to systemic hypoxia, and not as a direct result of the trauma with primary brain damage.

**Table 2:** Treatment outcomes.

Admitted Patients (N = 7)	
Stay on ward (in days) ^2^, mean ± SD (range)	3.43 ± 5.65 (0–16)
Stay on ICU (in days), median (IQR, range)	0 (3, 0–25)
LOS (in days) ^1,2,^ median (IQR, range)	3 (17, 1–41)
Number of surgical interventions ^2^, median (IQR, range)	0 (1, 0–4)
Need of blood transfusion ^2^, N (%)	1 (16.7)
GOS at discharge, N (%) ^1,3^	
1 Dead	0
2 Vegetative state	0
3 Severe disability	0
4 Moderate disability	3 (21.4)
5 Good recovery	10 (71.4)
Unknown	1 (7.1)

^1^ Abbreviations: LOS length of stay, GOS Glasgow Outcome Score. ^2^ Stay on the ward, ICU, LOS, and the number of surgical interventions were determined for patients that were admitted to the hospital through the ED (*n* = 7). Patients that were dismissed (*n* = 6) or transferred (*n* = 1) were not included in the analysis. ^3^ Glasgow outcomes scores are presented for the total study population (N = 14).

**Table 3:** Comparison of characteristics.

	Pre and Post Max Verstappen’s Championship		
	2015–2021 (N = 6)	2022–2023 (N = 8)	*p*-Value
Age, mean ± SD (range)	12.50 ± 2.88 (9–17)	10.75 ± 4.17 (4–16)	0.397 ^1^
Gender, N (%)			
Male	6 (100)	6 (75)	0.473 ^3^
Female	0	2 (25)	0.473 ^3^
Driving experience, N (%)			
Unexperienced	2 (33.3)	0	**0.048** **^3^**
Experienced	0	5 (62.5)	**0.048 ^3^**
Unknown	4 (66.7)	3 (37.5)	
Safety devices usage, N (%)			
Helmet	2 (33.3)	7 (87.5)	0.091 ^3^
Neck brace	0	5 (62.5)	**0.031 ^3^**
Seatbelt	2 (33.3)	2 (25)	1.000 ^3^
Protective clothing	2 (33.3)	3 (37.5)	1.000 ^3^
ISS, mean ± SD (range)	8.00 ± 9.25	9.00 ± 8.78	0.840 ^1^
Admission after ED	3 (50)	4 (50)	0.459 ^4^
Stay on ward ^5^, mean ± SD	6 ± 8.2	1.5 ± 1.3	0.341 ^1^
Stay on ICU ^5^, mean ± SD	9 ± 13.89	0.75 ± 1.5 (median 0, IQR 2.25)	0.329 ^2^
LOS ^5^, mean ± SD	20.67 ± 19.55	2.25 ± 0.96	0.109 ^1^
N of surgical interventions ^5^, median (range)	1 (1–4)	0	**0.018 ^2^**

^1^ *T*-test for independent samples ^2^ Mann–Whitney U test. ^3^ Fisher’s exact test. ^4^ Chi-square test. ^5^ Statistics for stay on ward, stay on ICU, LOS, and N of surgical interventions were calculated for patients admitted to the hospital: respectively 3 and 4 patients within the cohorts. Bold values are significant (*p* < 0.05).

## Data Availability

The data included in this study are not publicly available due to privacy reasons but may be made available upon reasonable request to the corresponding author.

## Abbreviations

The following abbreviations are used in this manuscript:

ED	Emergency department
DNTR	Dutch Nationwide Trauma Registry
ASA	American Society of Anesthesiologists
AIS	Abbreviated Injury Scale
ISS	Injury Severity Score
GCS	Glasgow Coma Scale
ICU	Intensive care unit
LOS	Length of stay
GOS	Glasgow Outcome Score
TBI	Traumatic brain injury
CT	Computed tomography
MRI	Magnetic Resonance Imaging
RTA	Road traffic accident
